# Gut microbiota supports male reproduction *via* nutrition, immunity, and signaling

**DOI:** 10.3389/fmicb.2022.977574

**Published:** 2022-08-18

**Authors:** Hui Cai, Xuanhong Cao, Dezhe Qin, Yundie Liu, Yang Liu, Jinlian Hua, Sha Peng

**Affiliations:** ^1^Shaanxi Centre of Stem Cells Engineering and Technology, College of Veterinary Medicine, Northwest A&F University, Shaanxi, China; ^2^State Key Laboratory for Molecular and Developmental Biology, Institute of Genetics and Developmental Biology, Chinese Academy of Sciences, Beijing, China

**Keywords:** gut microbiota, testis, male fertility, gut-testis axis, probiotics

## Abstract

Gut microbiota (GM) is a major component of the gastrointestinal tract. Growing evidence suggests that it has various effects on many distal organs including the male reproductive system in mammals. GM and testis form the gut-testis axis involving the production of key molecules through microbial metabolism or *de novo* synthesis. These molecules have nutrition, immunity, and hormone-related functions and promote the male reproductive system *via* the circulatory system. GM helps maintain the integral structure of testes and regulates testicular immunity to protect the spermatogenic environment. Factors damaging GM negatively impact male reproductive function, however, the related mechanism is unknown. Also, the correlation between GM and testis remains to be yet investigated. This review discusses the complex influence of GM on the male reproductive system highlighting the impact on male fertility.

## Introduction

Gut microbiota (GM) is an indispensable regulator of host metabolism, immunity, and endocrine functions. Its composition, abundance, metabolites, and signaling pathways significantly impact organ development starting from the local intestine to distal organs. The metabolic outcomes of GM determine key processes like lipid and bile metabolism, vitamin and short-chain fatty acids production, pathogens resistance, DNA expression and detoxification (Walter and Ley, 2011). The genetic and chemical diversity of GM is far greater than that of the host genome as GM includes trillions of symbiotic bacteria, virus, and fungi in the intestine (Lam et al., 2022; Schupack et al., 2022). As for bacteria, intestinal microenvironment is mainly conducive to the growth of six major bacterial phyla, including *Firmicutes*, Bacteroides, Proteobacteria, Actinomycetes, Verrucomicrobia, and Fusobacterium (Eckburg et al., 2005). Among them, *Bacteroides* and *Firmicutes* account for >90% (Qin et al., 2010), and their proportions change dynamically during different stages of animal life. Although a large part of GM remains conserved, evidence suggests that the microbial abundance of GM changes dynamically at the species level depending on the host’s age or health conditions (Yatsunenko et al., 2012). These features allow GM to work much better in different phases/health conditions. Although GM is dynamic, it has some basic functions regulating immunity, metabolism, and nervous system impacting the general physical and mental health of the host (Adak and Khan, 2019).

**FIGURE 1:**
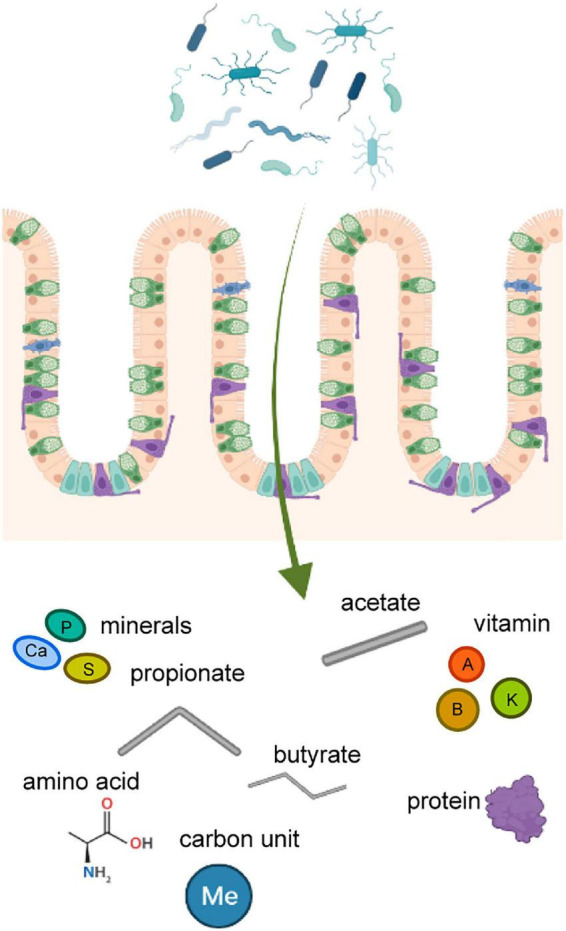
The nutrient substance produced by gut microbiota. Products secreted by gut microbiota in the intestine affect the distal parts of body through the circulatory system. These substances include amino acids, proteins, vitamins, minerals, fatty acids, and so on.

In GM mediated digestion of nutrients, the main end product of carbohydrates is short-chain fatty acids (SCFAs), which play a role in the metabolism and circulation of glucose and lipid. Three kinds of SCFAs, propionate, acetate, and butyrate, have roles in maintaining intestinal integrity and relieving inflammation (Morrison and Preston, 2016). Amino acids and short peptides produced in the digestive tract after proteolysis are used by GM to synthesize other kinds of proteins. Moreover, GM can enzymatically decompose protein products to generate energy or produce signaling molecules to regulate the physiological state of the host (Figure 1; Nicholson et al., 2012). The GM balance is very critical to the homeostasis of the host’s immune system. Beneficial strains strengthen the tight junctions of the intestine. In case of disturbed GM, immune responses are generated in the local intestine, which can gradually advance to inflammatory bowel disease (IBD). The integrity of the intestine is impaired by inflammation induced by bacteria-produced lipopolysaccharides (LPS) and inflammatory cytokines, which can circulate and spread to other organs (Ulluwishewa et al., 2011).

The health status or balance of the GM also affects the development and health of the male reproductive system of in mammals (Martinot et al., 2021). This effect could be positive or negative in nature (Guo et al., 2020; Liu et al., 2022). Certainly, the reproductive ability of male animals directly determines the survival and reproduction of organisms, which can become vulnerable at the time of imbalanced GM. Also, the testicles, wrapped in the scrotum outside the body cavity, are easily vulnerable to changes in the internal and external environment. Heat and cold stress, hormone levels and endocrine disruptors, dietary structure, exercise, growth and development, and congenital factors all have effects on the function of testis (Belloc et al., 2014; Tian et al., 2019; Qin et al., 2021). According to the World Health Organization (WHO), 15% of couples worldwide struggle with infertility, of which, 50% of cases of infertility are due to men having troubles such as varicoceles and azoospermia (Jensen et al., 2017; Wu et al., 2021).

The growth of the testis relies on germ and somatic cells. A mature testis produces sperm. Spermatozoa accumulate in the epididymis and are then discharged out of the penis through the deferens to complete the jaculation process. This process requires nutrients such as water, amino acids, lipids, carbohydrates, vitamins, and minerals. Germ cells achieve differentiation and maturation through the process of exchanging nutrients and metabolic wastes with Sertoli cells (Dance et al., 2015). In addition, the erection of the penis requires stimuli from various gas signaling molecules, which are majorly produced by cyclic metabolism in GM (Zmora et al., 2019). Recent studies found a small number of bacteria in testicles are similar to gut bacteria and semen can regulate certain male reproductive diseases (Altmae et al., 2019; Godia et al., 2020). From the perspective of the male reproductive system, the testes and penis protect the germ cells, while GM absorbs and metabolizes nutrients to ensure the functioning of male reproductive organs. This review aims to expound on the regulation of GM that in multiple ways promotes the growth and development of the male reproductive system.

## Gut microbiota supports testis by metabolizing nutrients

Testes cannot *de novo* synthesize nutrients. The blood vessels in the testis transport nutrients, including those synthesized or metabolized by GM, from the digestive system to the testicular interstitium *via* the convoluted seminiferous tubules through Sertoli cells and their intercellular connections. Nutrients such as vitamins and minerals synthesized or metabolized by GM are essential for testes (Table 1). The changed microbiota may disturb the original nutritional structure and function of the testis.

**TABLE 1:** The summary of gut microbiota produced nutrients affecting the male reproductive system.

Nutrients	Function in the male reproductive system	Main bacteria producer
Vitamin A	Promotes spermatogonial stem cells differentiation into sperms	*Escherichia coil*, *Clostridia* (Stacchiotti et al., 2021)
Folic acid	Promoting germ cell differentiation, resistance of oxidative stress and inflammation; prevention of hyperhomocysteinemia.	*Lactobacillus*, *Bifidobacterium*, *Acidobacillus* (Kadry and Megeed, 2018; Wu et al., 2022)
Calcium	Improves sperm motility and sperm capacitation; activates acrosome reaction, and signal transduction in germ cells	*Bifidobacteria*, *Lactobacillus* (D’Amelio and Sassi, 2018)
Vitamin K	Resistance against inflammatory response; promotes serum testosterone and the blood-testis barrier	*Bacteroides fragilis* (Stacchiotti et al., 2021)

### Vitamin A

Vitamin A is an indispensable nutrient for the reproductive system and embryonic development. One of the metabolic forms of vitamin A is retinoic acid (RA), which prompts the stagnant spermatogonial stem cells in the G0/G1 stage in the embryo to initiate meiosis for differentiation into mature sperms. Vitamin A deficiency leads to the failure of type A spermatogonial stem cells differentiation into type A1; the stratified squamous keratinizing epithelium replaces epithelia of the prostate, epididymis, and seminal vesicle, slowing sperm production (Clagett-Dame and Knutson, 2011). In humans and mice, the expression of two genes related to spermatogenesis (*Stra8* and *Rec8*) is promoted by RA. Without the expression of *Stra8*, undifferentiated spermatogonia are difficult to accumulate and differentiate, which causes the failure of meiosis. In Sertoli cells, RA binds to retinoic acid receptor (RAR) recruiting retinoid X receptors (RXRs), which promotes the transcription process. Interference of RAR or RXRs in Sertoli cells blocking the RA-RAR/RXR signaling causes the failure of the blood-testis barrier (BTB), which forbids sperms to mature and release from Sertoli cells (Schleif et al., 2022). GM plays an important role in regulating the intestinal absorption and metabolism of vitamin A. Proteins produced by *Escherichia coli* like RXRs and farnesoid X receptors have been linked to the transport of vitamin A to intestinal cells, where retinal dehydrogenase (RALDH) converts retinal into RA. Also, *Clostridia* directly modulates the RA concentration. Moreover, GM inhibits the activity of the cytochrome P450 (CYP) family of protein, which can degrade vitamin A. In addition, intestinal microbial enzymes promote the production of retinoic acid from b-carotene (Stacchiotti et al., 2021). In sheep, diet-induced metabolic disorders lead to the imbalance of GM reducing the production of bile acids and the absorption of vitamin A, a kind of fat-soluble vitamin. Consequently, it significantly increases the ratio of undifferentiated spermatogonia in the testis but decreases the number of mature sperms (Zhang et al., 2022).

### Folic acid

Folic acid (vitamin B9) is necessary for DNA and RNA synthesis and methylation. It can affect chromatin structure by affecting histone methylation which is necessary for cell division. Folic acid in the diet improves semen quality and testicular tissue structure, especially if the animal is exposed to reproductive toxic substances. Folic acid helps germ cells to resist oxidative stress and inflammation to prevent DNA damage and apoptosis. Also, it protects the proliferation and differentiation of germ cells from the accumulation of oxidative substances (Rad et al., 2021). Methylenetetrahydrofolate reductase (MTHFR) is one of the key enzymes in folic acid metabolism, which participates in the biosynthesis of tetrahydro folic acid (THFA) and vitamin B12 and can re-methylate homocysteine to methionine, an essential amino acid. The low levels of these two vitamins lead to hyperhomocysteinemia, a disease related to the failure of *in vitro* fertilization (IVF) and decreased sperm density, vitality, and DNA integrity. The circulating homocysteine and degree of oxidative stress are positively correlated (Fowler, 2005). Human *MTHFR* gene polymorphisms 677CT and 1298AC can cause a 70% reduction in folate metabolism and hyperhomocysteinemia. *MTHFR* 677T allele is an important factor for male infertility in Asia. Folic acid treatment for 3 months can significantly alleviate the semen oxidative stress due to *MTHFR* 677TT gene carriers, and decrease malondialdehyde and sperm DNA breakage index, improving the natural pregnancy rate and live birth rate (Huang et al., 2020). Folic acid is obtained mainly from dietary supplements and bacterial synthesis. THFA is synthesized by intestinal bacteria from GTP, erythrose 4-phosphate, and phosphoenolpyruvate, which is directly absorbed through the proton-coupled folate transporter of colon cells and distributed through the circulatory system. Metagenomic analysis showed that *Bacteroides fragilis* and *Prevotella copri* of Bacteroidetes, *Clostridium difficile*, *Lactobacillus plantarum*, *L. reuteri*, *L. delbrueckii* ssp., *bulgaricus* and *Streptococcus thermophilus* of Firmicutes, part of *Bifidobacterium* spp. of Actinobacteria, *Fusobacterium varium* of Fusobacteria, and *Salmonella enterica* of Proteobacteria genera play a role in THFA synthesis (Yoshii et al., 2019). In GM, *Lactobacillus* and *Bifidobacterium* of GM are the main folic acid-producing and metabolizing bacteria (Wu et al., 2022). The production of folic acid can be detected in the culture system of human fecal microbiota *in vitro*. A study showed that oral *Lactobacillus* or *Acidobacillus* in cadmium-poisoned mice reduced testicular cadmium poisoning and promoted germ cell formation, which is a similar effect to folic acid supplementation (Kadry and Megeed, 2018).

### Calcium

Calcium plays a decisive role in the fertilization process. It regulates sperm motility in mammals, which directly determines the occurrence of sperm-egg fusion. The process of sperm capacitation is dependent on the activation of the calcium ion channels on the sperm flagellum for sperm motility into the female reproductive tract (Vyklicka and Lishko, 2020). This chemotactic behavior determines acrosome reaction, including hyper activated motility (HAM) like progressive motility and flagellar asymmetric motility. For the acrosome reaction, the sperm needs a sustained increase in intracellular Ca^2+^ levels until F-actin is released from the plasma membrane. Ca^2+^ induces HAM by regulating F-actin, and its influx is mainly controlled by CatSper, which is a sperm-specific Ca^2+^ channel. Ca^2+^ influx can also produce cAMP through a cascade signal reaction promoting active protein kinase A (PKA) causing protein tyrosine phosphorylation. Ultimately, signal transduction in sperm is promoted. Calcium ions accumulate in the epididymis and prostate fluid against the concentration gradient, which is 2–3 times higher than that of the circulatory blood levels (Finkelstein et al., 2020). Blood calcium concentration is sustained by the dissolution of calcium salts *via* osteoclasts in bones. GM is the main regulator of mammalian bone mass, which regulates Ca^2+^ levels in the reproductive system by regulating the conversion between blood and bone calcium. In GM, *Bifidobacteria* and *Lactobacillus* affect the absorption of food calcium. The short-chain fatty acids (SCFAs) in the colon are the regulator of bone cell metabolism. GM produces SCFAs by decomposing dietary fiber. SCFAs reduce the formation of calcium phosphate and promote calcium absorption by lowering the intestine Ph (D’Amelio and Sassi, 2018). A study showed that the levels of IL-6, RANKL, and TNF-α in bone tissues decreased in germ-free mice lowering the number of osteoclasts than SPF mice. SCFAs increase calcium transport through regulations of signaling pathways. Additionally, SCFAs promote the synthesis of serotonin (5-HT), which interacts with bone cells *via* the activation of 5-HT1B receptors on pre-osteogenic cells to inhibit the proliferation of osteoblasts and reduce the formation of bone calcium. This ensures the blood calcium content (Sjogren et al., 2012). A study in GF (germ-free) mice showed an increase in bone mass, while the number of osteoclasts on the surface of bone decreased lowering the concentration of free Ca^2+^. Re-colonization of the GM in GF mice could normalize the bone mass (Ding K. et al., 2020). This modulating effect of GM on the calcium salt status either promotes or inhibits the survival and motility status of sperm.

### Vitamin K

There are two sources of natural vitamin K, plant-derived phylloquinone (vitamin K1), and menaquinone (vitamin K2 or MK-n) produced by microorganisms. In mammals, GM synthesizes menaquinone and transports it through the circulatory system. Vitamin K1 must be converted into vitamin K2 to play important physiological functions such as blood coagulation, fibrinolysis, and bone homeostasis. A vitamin K-rich diet can improve the inflammatory resistance ability of the testes. It can also upregulate the cholesterol and steroid hormone synthase genes, such as *Cyp11a*, thereby increasing the concentration of serum testosterone. In the testicular inflammation rat model induced by LPS, inflammatory mediators such as Nuclear Factor kappa B (NF-κB) and pro-inflammatory factors reduced the transcriptional activity of steroidogenic factor 1 and cyclic AMP response element-binding protein that regulate *Cyp11a*. Consequently, the reduced expression of *Cyp11a* decreased inhibited the synthesis of testosterone in the testis. In all, vitamin K inhibited the activation of NF-κB, increased the expression of *Cyp11a* after LPS treatment, and reduced the inhibitory effect of inflammatory stimulation on testosterone synthesis (Takumi et al., 2011). Vitamin K, as a cofactor, helps γ-glutamyl carboxylase (GGCX) to carboxylate glutamic acid residues into γ-carboxyglutamic acid residues, which then activates vitamin K-dependent proteins. GGCX in testis may promote vitamin K-dependent γ-carboxylation of androgen receptor in Sertoli cell, which helps maintain the BTB structure, and facilitates the development of germ cells and sperm release (Shiba et al., 2021). In idiopathic non-obstructive azoospermia (iNOA) patients, vitamin K epoxide reductase complex subunit 1 (VKORC1), the substrate of vitamin K cycle metabolism, was found abnormally deleted in Leydig cells and extracellular matrix (Alfano et al., 2019). In addition, the relative proportion of vitamin K and D also significantly impact calcium metabolism affecting the development and motility of sperm (Khalil et al., 2021). Human or mice GM can add or reduce the side chain of dietary supplement vitamin K precursor to remodel to menaquinone 4, 10, 11, and 12 for further utilization (Ellis et al., 2021). GM like *Bacteroides fragilis* can produce vitamin K, mainly the menaquinones. MK4 promotes the genes related to testosterone synthesis. Also, MK7 works with Vitamin D to regulate the level of calcium (Stacchiotti et al., 2021).

## Gut microbiota regulates the immune microenvironment of testis

**FIGURE 2:**
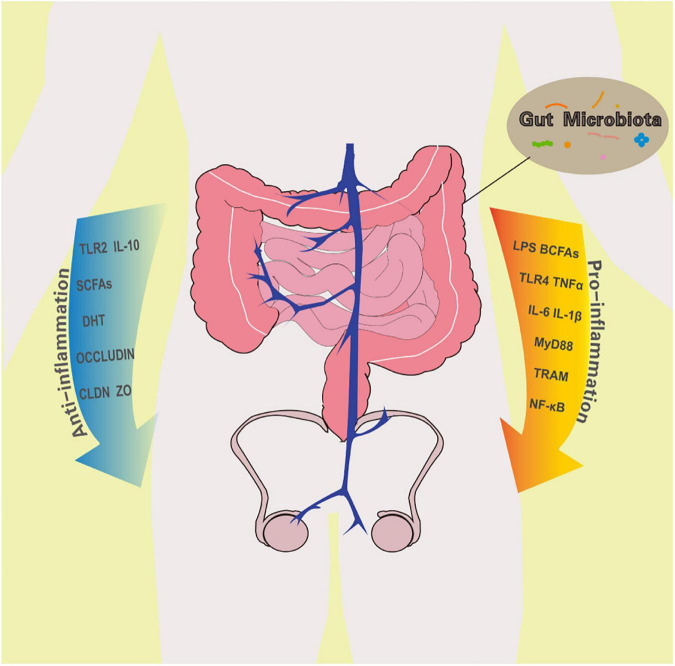
The effect of gut microbiota on testes immune privileged microenvironment. A healthy GM promotes anti-inflammatory cells and factors. However, when abnormal bacteria multiply in large numbers, they increase the concentration of pro-inflammatory molecules in the intestine and body fluids. Both positive and negative changes in GM affect the status of the testicular immune microenvironment. TLR2, Toll-like receptor 2; IL-10, Interleukin-10; SCFAs, short chain fatty acids; DHT, dihydrotestosterone; CLDN, claudins; ZO, Zona occludens; LPS, lipopolysaccharide; BCFAs, branched chain fatty acids; TLR4, Toll-like receptor 4; TNFα, tumor necrosis factor α; IL-6, Interleukin-6; IL-1β, Interleukin-1β; MyD88, myeloid differentiation factor 88; TRAM, translocation associated membrane protein; NF-κB: nuclear factor kappa B.

Testes are immune privilege organs. Notably, the male haploid germ cells are not produced until the time of puberty, a long time after birth, which makes these new cells prone to the self-immune system (Qu et al., 2020). Therefore, these germ cells, which are self-antigens, are isolated from the environment to prevent attacks from the immune system. The seminiferous tubules are surrounded by a basement membrane, which is composed of supporting cells and intercellular connections in the blood-testis barrier, specialization of basal exoplasm, and muscle-like tubule cells. The seminiferous tubules create independent cavities, which block the attack from the immune system. Sertoli cells also phagocytose and digest apoptotic germ cells and their remnants to prevent autoimmunity. Androgens synthesized by interstitial cells, corticosterone secreted by testicular macrophages, and prostaglandins, activin, and 25-hydroxycholesterol present in the interstitium inhibit the function of macrophages in the testis. The secretion of corticosterone induces the differentiation of macrophages into immunosuppressive M2 type, promotes the secretion of anti-inflammatory cytokine IL-10, inhibits the expression of TNFα, IL-6, and other pro-inflammatory factors, and reduces the level of the immune response (Wang et al., 2017). The regulatory T lymphocytes (Treg cells) present in the testis upregulate the anti-inflammatory factors IL-10, IL-35, and TGF-β, creating an immunosuppressive microenvironment. A higher number of effector T cells over Treg cells in the testis disturbs the immune-suppressed environment and the autoimmune response is activated (Jacobo, 2018). Although GM promotes maintenance of the immune privileged microenvironment of testis in multiple ways, it can also break it in adverse situation (Figure 2).

### Gut microbiota and peripheral immunity

The crosstalk between GM and the peripheral immune system influences the balance of pro- and anti-inflammatory cells and maintains the tolerance of the immune exemption department in testis. *Bacteroides Fragilis* produces Polysaccharide A, activates TLR2 signal to induce the production of Foxp3 + Treg cells, promotes the secretion of anti-inflammatory factor IL-10, and inhibits the effect of pro-inflammatory Th17 cells. All this enhances the organ resistance against inflammation (Round and Mazmanian, 2010). SCFAs, an important product of gut microbes, especially butyrate, can facilitate peripheral naive CD4 + T cells of extrathymus to differentiate into Foxp3 + Treg cells (Arpaia et al., 2013). Also, butyric acid promotes the differentiation of M2 macrophages (Ji et al., 2016). SCFAs inhibit the NF-κB pathway by inhibiting lipopolysaccharide-induced macrophages to produce nitric oxide and pro-inflammatory cytokines TNFα, IL-1β, and IL-6 while promoting the secretion of IL-10 (Liu et al., 2012). Finally, the produced immune cells reach the testis *via* mesenteric the lymph system, hepatic portal vein and testicular artery, and affect the immune microenvironment of the testis. In the case of disturbed GM, the secretion of pro-inflammatory factors increases activating macrophages and dendritic cells in the testis. When these innate immune cells enter the epididymis, sperms are recognized as non-self substances attacked, affecting their survival and function (Zheng et al., 2021).

### Gut microbiota and androgen

GM regulates the development of gonads through the gut-brain axis, promotes androgen synthesis, and protects the testicular immune tolerance. Androgens ensure the level of Treg cells, inhibit the proliferation of NK cells, and also protect the structure of BTB to prevent pathogenic substances (Kabbesh et al., 2021). A study showed that gut microbes have a strong ability to promote testosterone levels. In adult mice, the level of dihydrotestosterone (DHT) in feces is >20 times higher than that in serum (Collden et al., 2019). Furthermore, compared with sterile mice, the normal concentration of free DHT in the intestine of normal mice was higher. Also, the levels of testosterone, serum gonadotropins luteinizing hormone (LH), and follicle-stimulating hormone (FSH) were higher in the testes of normal mice or mice colonized with probiotics than those in sterile mice. In addition, genes controlling the testosterone production in GF mice such as *Hsd3b1*, *Hsd17b11*, *Cyp11a1*, and *INSL3* were down regulated (Al-Asmakh et al., 2014). GM-produced LPS and pro-inflammatory factors degrade testicular IkB and promote the expression of upstream kinase IKK, which promotes nuclear translocation of NF-κB and inhibits transcription. Phosphorylated NF-κB inhibits the transcription of SF-1 and CREB in testis decreasing the expression of steroid producing gene Cyp11a and testosterone levels. This process can be reversed by increasing the colonization of GM synthesizing vitamin K (Takumi et al., 2011).

### Gut microbiota and blood-testis barrier

A healthy GM improves the integrity of the BTB by upregulating intercellular connections and reducing permeability. The BTB is composed of Sertoli cells and adhesion junction (AJ) and tight junction (TJ) proteins between the cells, such as occludin, claudins (CLDN), JAM, Zona occludens (ZO-1, ZO-2, ZO-3) (Mruk and Cheng, 2015). GM promotes the development of Sertoli cells and their tight junctions, thereby ensuring the formation of seminiferous tubules and the safety of the microenvironment. In 15–16 days old prepubertal mice, SPF mice showed more complete seminiferous tubule development and lumen formation than GF mice. Due to underdeveloped Sertoli cells and low quantity, the lumen of the seminiferous tubules of GF mice was more atresia, showing no attachment of mature luminal co-germ cells. The expression of adhesion links and tight junction proteins, such as occludin, ZO-2, and E-cadherin, was also lower in GF mice. The re-colonization of probiotics in the intestines of GF mice improved the above situation. Due to the loss of Sertoli cells and their intercellular connections, the BTB of GF mice showed higher permeability than SPF mice. The Evans Blue (EB) perfusion test showed higher fluorescence intensity in the seminiferous tubules of GF mice, while after probiotics colonization, the fluorescence only appeared in the interstitium outside the seminiferous tubules (Al-Asmakh et al., 2014). The colonization with normal GM promotes the development of the BTB by improving the secretion of androgen. Testosterone binds to the testosterone receptor on Sertoli cells and promotes the expression of Claudin3 protein in Sertoli cells to increase the tightness of the BTB (Meng et al., 2005). Bacterial translocation induced by abnormal intestinal permeability leads to oxidative stress, activates testicular LPS/TLR4, and transfers NF-κB and mitogen-activated protein kinase to the nucleus through the MyD88 and TRAM pathways. This activates the innate immunity damaging testicular endothelium and the BTB (Wang and Xie, 2022).

### Gut microbiota and testicular immune environment

The altered composition of GM can change gut permeability and immune status through its metabolites, endotoxins, and pro-inflammatory factors, thereby, affecting the immune environment of testis and damaging the reproductive system. A study showed that boars with highly abnormal sperm rates and lower semen utilization contained higher plasma endotoxin and pro-inflammatory factors such as TNF- α and IL-6, and lower anti-inflammatory factor such as IL-10 (Guo et al., 2020). Also, the concentration of fecal branch chain fatty acids (BCFAs), and the markers of proteolysis in the colon were significantly higher than that in boars with high-quality semen. Meanwhile, zonulin and diamineoxidase, which destroy the integrity of the intestine, were also higher in the plasma of boars with low-quality semen. Eventually, the study found that *Sphingobium*, a genus of bacteria that destroys the integrity of the intestine, was enriched in the GM of boars with low-quality semen. The abundance of *Sphingobium* had a strong positive correlation with plasma endotoxin. simultaneously, the abundance of gram-negative *Proteobacteria* in the intestine of boars also increased with low semen utilization. BCFAs, the product of abnormal protein breakdown by *Proteobacteria*, showed higher enrichment in the feces of boars with poor semen quality, which is an indicator of increased intestinal permeability. *Proteobacteria* use amino acids to produce BCFAs while other toxic metabolites are produced in the process. This suggests that higher levels of *Sphingobium* and *Proteobacteria* in the intestine may cause inflammatory responses decreasing semen quality. Increased intestinal permeability promotes LPS leakage into the blood, activates Toll-like receptors, and triggers the immune system to produce IL-6 and TNFα and other pro-inflammatory factors causing immune attacks on the testis (Vaarala et al., 2008; El-Baz et al., 2021). Eventually, the sperm cell membrane in such boars is damaged by lipid peroxidation, the vitality is reduced, and the damage to sperm DNA increases. Also, testosterone synthesis is reduced lowering reproductive ability.

## Gut microbiota regulates testis by releasing signaling molecules

The growth, development, and functional regulation of the male reproductive system are also affected by various signaling molecules. For example, 5-hydroxytryptamine (5-HT, serotonin), γ-aminobutyric acid (GABA), and dopamine can regulate androgen levels and the process of sperm capacitation. Nitric oxide (NO), carbon monoxide (CO), hydrogen sulfide (H_2_S), and sulfur dioxide (SO_2_) are important signal molecules synthesized from arginine, glycine, and cysteine, respectively (Table 2). These activate guanylate cyclase to produce cGMP, which regulates vascular smooth muscle cell relaxation, hemodynamics, neurotransmission, and cell metabolism through cGMP-dependent protein kinases. H_2_S is also an important regulator of nerve function and endothelium-dependent relaxation, regulating membrane KATP channel stimulation and intracellular cAMP signal transmission. In addition, NH_3_ is the main product of amino acid catabolism in bacteria and profoundly affects the function of neurons and the vascular system through glutamine-dependent inhibition of NO synthesis (Li et al., 2009).

**TABLE 2:** The summary of GM secreted signaling molecules regulating the male reproductive system.

Signaling molecules	Regulation of the male reproductive system	Main bacteria producer
GABA	Promotes sperm capacitation and acrosomal reaction; reduces the excessive activation of sperm; increases libido and sexual behavior	*Bacteroides*, *Parabacter* and *Escherichia coli* (Strandwitz et al., 2019)
5-HT	Balances androgens; reduces the weight and volume of the testis; inhibits ejaculation	*Escherichia coli*, *Streptococcus*, *Enterococcus*, *Bacillus*, Spore-forming microbes, *Clostridium ramosum* and *Corynebacterium* spp. (Yano et al., 2015; Mandic et al., 2019; Liu et al., 2020)
NO	Induces penis erection	*Lactobaillus* spp., *Bifidobacterium* spp., *Staphylococcus aureus*, *Bacillus* spp. (Dai et al., 2015)
H_2_S and SO_2_	Induce penis erection	*Desulfovibrio, Desulfobacter, Desulfobulbus* and *Desulfotomaculum* (Gibson et al., 1993; Ran et al., 2019)
LH, FSH and T	Promote testicular cell growth and function; support gonadal development and reproductive function	*Prevotellaceae*, *Cytophagaceae*, *Fibrobacteriaceae*, *Sphingobacteriaceae*, *Idiomarinaceae*, etc. (Markle et al., 2013)

### GABA and 5-HT

GM has been shown to produce various neurotransmitters, such as GABA, 5-HT, dopamine, and norepinephrine by metabolizing proteins and amino acids or by *de novo* synthesis (Dai et al., 2015). Experiments in mammals show that a large number of neurotransmitters molecules produced by GM play a role in maintaining and changing the physiological functions of animals (Strandwitz, 2018; Huang and Wu, 2021). The presence of 5-HT in the testis balances the production of androgens. In rat interstitial cells, 5-HT binds to 5-HT2 receptors to stimulate the secretion of corticotropin-releasing factor (CRF), which inhibits the synthesis of cAMP and gonadotropin-induced androgen (Tinajero et al., 1993). 5-HT (four times a day, 10 mg/kg) injected into the abdominal cavity of rats reduced the weight and volume of the testis, and lowered the concentration of inhibin and serum testosterone (Hedger et al., 1995). Also, 5-HT inhibits ejaculation and adjusts penile flaccidity and detumescence *via* the control of vascular resistance, blood pressure, hemostasis and platelet function. 5-HT binding to 5-HT2C and 5-HT1B receptors increases ejaculatory latency and delays orgasm, while 5-HT binding to 5-HT1A receptor decreases ejaculatory latency. The testis itself can produce endogenous 5-HT, while the rest is mainly used from the peripheral circulation (Berger et al., 2009). The gut is the main source of 5-HT; >90% of the total 5-HT is gut-derived, which is transported to the whole body through platelets. Enterochromaffin cells (ECs), mucosal mast cells, and myenteric neuron cells mainly synthesize 5-HT in the intestine. Studies have shown that nearly 10% of ECs synthesis peripheral 5-HT rely on GM. The concentration of serum 5-HT in adult GF mice decreased, and correspondingly, the concentration of 5-HT in the colon and feces decreased significantly. Spore-forming microbes (Sp) from the healthy mouse and human microbiota promote local and peripheral 5-HT concentration through its metabolites to promote the expression of tryptophan hydroxylase 1 (*Tph1*), an important gene for 5-HT synthesis in ECs (Yano et al., 2015). Cellular components of *Clostridium ramosum* have also been shown to stimulate host ECs to secrete 5-HT and modify the colonic stem cells to differentiate into lineages that secrete 5-HT (Mandic et al., 2019). Some bacteria in culture, including *Corynebacterium* spp., *Streptococcus* spp. and *Escherichia coli*, were reported to synthesize 5-HT (Yano et al., 2015). Damage GM induces local inflammation, which lowers the number of 5-HT transporters (Stasi et al., 2019). A study showed that male Brandt’s voles reared in high density stress environments exhibited a higher abundance of *Streptococcus* and *E. coli* in the intestine, which possibly increases the serum cortisol and 5-HT concentrations. Both of these increased the serum testosterone levels of Brandt’s voles *via* the hypothalamic-pituitary-gonadal axis making the animal more aggressive (Liu et al., 2020).

The GM genome metabolism model showed that *Bacteroides*, *Parabacter* and *E. coli* actively express GABA. Also, the isolation and culture of *Lactobacillus* and *Bifidobacterium* in the intestine could produce GABA. The GABA concentration is related to the process of sperm capacitation in the vagina. GABA promotes the tyrosine phosphorylation of sperm protein, which is an indicator of sperm capacitation. GABA also promotes the acrosome reaction, which is inhibited by selective GABA receptor antagonists (Kurata et al., 2019). A study in hamsters showed that GABA reduces the excessive activation of sperm by inhibiting the binding of 5-HT to 5-HT2 receptors, thereby co-regulating sperm activation with 5-HT (Fujinoki and Takei, 2017). GABA can also regulate the sexual behavior of male mammals. Treatment with Moxidectin, an anthelmintic drug, in rats lowered their libido and sexual behavior by reducing GABA secretion, which hindered penile erection (Rodrigues-Alves et al., 2008).

### Nitric oxide and hydrogen sulfide

Arginine amino acid has nutritional effects on male reproductive function. Although bacteria in the small intestine can decompose arginine and affect the use of arginine by the reproductive system, some bacteria such as *Lactobaillus* spp., *Bifidobacterium* spp., *Staphylococcus aureus*, *Bacillus* spp. affect the NO production *via* arginine metabolism (Dai et al., 2015). NO synthesizing bacteria Bacillus and Paenibacillus were found in the GM of obese girls, and the NO synthesis was positively correlated with the level of FSH (Li Y. et al., 2021). Physiological levels of NO, a signaling molecule, also play an important role in the male reproductive system. In the brain, NO promotes the release of neurotransmitters to maintain libido and the secretion of luteinizing hormone-releasing hormone (LHRH) and GnRH to increase sex hormone levels. In testis, NO dilates blood vessels, allowing the testes to regulate local temperature. In the reproductive system, NO is released at the nerve endings of the cavernous body to activate guanosine cyclase. Activated guanylate cyclase produces cGMP to relax the vascular smooth muscle congesting the corpus cavernosum which leads to penis erection (Gratzke et al., 2010). H_2_S can also act as a physiological vasodilator, which directly affects erectile function. A study showed an increase in penis length after H_2_S injection into the penile cavernous body; the efficiency of penis lengthening was similar to the effect of 20 μg prostaglandin E1 (D’Emmanuele di Villa Bianca et al., 2011). Intestinal sulfate-reducing bacteria (SRB) such as *Desulfovibrio* spp., can use H_2_, lactic acid, and acetate as electron donors, and sulfate or sulfite as electron acceptors to produce H_2_S. There are also some bacteria in large intestines, such as *E. coli*, *Salmonella enterica*, *Clostridium* spp., and *Enterobacter aerogenes*, that can metabolize sulfur-containing amino acids to produce H_2_S (Gibson et al., 1993; Ran et al., 2019).

### Sex hormone

The type and abundance of gut microbes can affect the level of sex hormones in animals. Bacterial overgrowth in the small intestine may trigger an increase in intestinal permeability and systemic circulation, and a decrease in serum testosterone, which impairs testicular function (Tremellen and Pearce, 2020). This effect may be achieved by interfering with the steroid cycle metabolism and affecting the hormone-HPG axis. The level of sex steroid hormones is related to the composition and diversity of gut microbes. Individuals with more diverse gut microbes have higher levels of sex steroids (Shin et al., 2019). Estrogens (such as estradiol), progesterone, and their receptor exist in male sexual glands maintain male fertility. In women, estrogen production requires GM-secreted β-glucuronidase to covert conjugated estrogens to deconjugated forms. The increase in the abundance of β-glucuronidase-producing bacteria can promote in the level of circulating estrogen. A study showed that the α diversity of GM negatively correlates with the concentration of estradiol and positively correlates with the proportion of estrogen metabolites in the urine of women, however, the same needs to be verified in men and male animals (Qi et al., 2021). As mentioned earlier, GM can alter the expression of steroid-producing genes HSD3β1, Cyp11a, etc., which changes the levels of sex hormones (Takumi et al., 2011; Ding N. et al., 2020). Compared with SPF male mice, GF male mice had lower serum levels of testosterone (T), LH, and FSH, however, colonizing their intestine with probiotics significantly increased the serum levels of these hormones (Al-Asmakh et al., 2014). A study in the O-PLS mice model showed that testosterone levels were positively associated with *Prevotellaceae*, *Cytophagaceae*, *Fibrobacteriaceae*, *Sphingobacteriaceae*, and *Idiomarinaceae*, and negatively associated with *Actinobacteria*, *Proteobacteria*, *Firmicutes* and *Verrucomicrobia* phylum (Markle et al., 2013). In adolescent males, the level of testosterone was found to be associated with *Adlercreutzia*, *Ruminococcus*, *Dorea*, *Clostridium* and *Parabacteroides* genus (Yuan et al., 2020). Besides, a part of GM converts androgen precursors into active androgens (Pernigoni et al., 2021). GM promotes the deglucuronidation of testosterone and DHT, and increases the levels of free testosterone and DHT, which contribute to the development of secondary sexual characteristics in male animals. GC-MS (Gas chromatography-tandem mass spectrometry) analysis revealed that the intestinal levels of free testosterone and DHT were higher in segments with a high microbial density such as the cecum and colon than in a low microbial density segment such as the proximal small intestine. The free DHT level in feces is >70 times higher than in serum. Compared to normal mice, the concentration of free DHT is much lower in the distal intestine of GF mice, which contained a lot of glucuronidated T and DHT (Collden et al., 2019). Transplantation of fecal microbes from high-fat diet mice were into the intestine of normal mice increased the intestinal abundance of *Bacteroidaceae* and *Prevotellaceae* in the transplanted mice decreasing the expression of the Hsd3β1 gene encoding DHT synthase in testis (Ding N. et al., 2020).

## Conclusion and perspectives

GM metabolizes nutrients in animals regulating their immune state. GM has great research value for its effect on far distal organs. Experimental and clinical evidence from different species indicate that the main ways through which microbiota affects the development and function of the reproductive system include: providing nutrients like SCFAs, vitamins, and minerals to transform the function and gene expression status of the reproductive system, regulating the testicular immune microenvironment, controlling physiological processes through signal transduction, and affecting hormone levels (Dai et al., 2015; Li et al., 2017; Li X. et al., 2021; Zhang et al., 2022). The metabolic processes of GM provide crucial nutrients such as vitamins and minerals to the reproductive system; and regulate the development and functions of testes to maintain their immune privileged state. GF animals, which had no microbial abundance in the gut, exhibited decreased testosterone levels and abnormal BTB structure than the normal ones (Al-Asmakh et al., 2014). An altered GM negatively affects the function of the testis under on various stresses or the influence of toxic substances (Liu et al., 2022). Instead of providing nutritional molecules and support to the reproductive system, the abnormal microbiota produces pro-inflammatory factors and creates an oxidative environment that disrupts the spermatogenic process in the testis (Tian et al., 2019; Ding N. et al., 2020; Zhao et al., 2020). The effect of GM on distal organs is a fascinating prospect that requires more research. It may also provide a new promising way to regulate reproduction. Improving dietary structure, recolonizing healthy fecal microbes, and supplementing health products like probiotics have been shown to alleviate infertility in men and male animals, which further proves that altering the composition of GM can regulate the physiological functions of the testis, or even reverse the alterations to the aging effect on reproductive system (Poutahidis et al., 2014; Xie et al., 2019). Studies have shown that the decreased number of germ cells and low-quality semen in high-fat diet male animals are largely induced by GM disturbances which cause an accumulation of harmful metabolites such as sphingosine. Remodeling their GM by feeding melatonin or transplanting alginate oligosaccharides-improved fecal microbiota effectively alleviates the above conditions (Hao et al., 2022a; Sun et al., 2022). Zhang C. et al. (2021), Zhang P. et al. (2021), and Hao et al. (2022b) also found that transplantation of fecal microbiota from mice supplemented with alginate oligosaccharide to mice treated with busulfan or streptozotocin (a type 1 diabetes inducer) could rescue germ cell loss and improve semen quality through metabolic pathways. Although GM metabolites have an impact on fertility, basic phenomena yet remain to be defined completely. The physiological changes and specific consequences of this phenomenon are difficult to quantify, track and locate in real-time. For now, it is unknown how many metabolites from the circulatory system pass the BTB directly affect the male reproductive system. Existing research trends indicate that using multi-omics technology can delineate the interactions between GM and the host organs/tissues (Tilocca et al., 2020). With the establishment of gene expression profiles and metabolomics, researchers can now locate the transverse spatial organization and longitudinal phase states of GM (Tropini et al., 2017; Mars et al., 2020). The intricate networks between GM as well as the breaking and rebuilding of microbial balance are other research challenges. In the following research, scholars need to pay attention to the effect of partial and/or the entire function of the GM on toward the reproductive capacity in males and design a series of microbial complex agents to promote or inhibit fertility without affecting normal health (Alfano et al., 2018). Research targeting the treatment and development of GM will generate more emphasis in the near future to improve the health status of humans and animals.

## Author contributions

HC wrote the manuscript. SP investigated and supervised the manuscript. XC and DQ designed the tables and figures and edited the manuscript. JH, YDL, and YL edited the manuscript. All authors read and approved the final version of the manuscript.
